# Evolution, comparative biology and ontogeny of vertebrate heart regeneration

**DOI:** 10.1038/npjregenmed.2016.12

**Published:** 2016-07-28

**Authors:** Celine J Vivien, James E Hudson, Enzo R Porrello

**Affiliations:** 1 School of Biomedical Sciences, The University of Queensland, Brisbane, QLD, Australia; 2 Centre for Cardiac and Vascular Biology, The University of Queensland, Brisbane, QLD, Australia

## Abstract

There are 64,000 living species of vertebrates on our planet and all of them have a heart. Comparative analyses devoted to understanding the regenerative potential of the myocardium have been performed in a dozen vertebrate species with the aim of developing regenerative therapies for human heart disease. Based on this relatively small selection of animal models, important insights into the evolutionary conservation of regenerative mechanisms have been gained. In this review, we survey cardiac regeneration studies in diverse species to provide an evolutionary context for the lack of regenerative capacity in the adult mammalian heart. Our analyses highlight the importance of cardiac adaptations that have occurred over hundreds of millions of years during the transition from aquatic to terrestrial life, as well as during the transition from the womb to an oxygen-rich environment at birth. We also discuss the evolution and ontogeny of cardiac morphological, physiological and metabolic adaptations in the context of heart regeneration. Taken together, our findings suggest that cardiac regenerative potential correlates with a low-metabolic state, the inability to regulate body temperature, low heart pressure, hypoxia, immature cardiomyocyte structure and an immature immune system. A more complete understanding of the evolutionary context and developmental mechanisms governing cardiac regenerative capacity would provide stronger scientific foundations for the translation of cardiac regeneration therapies into the clinic.

## Introduction

In contrast to the extremely limited regenerative capacity of the adult human heart, certain fish, amphibians and neonatal mammals can fully recover cardiac function after injury. The sparse distribution of regenerative capacity across the animal kingdom appears to depend on the proliferative properties of cardiomyocytes. However, the mechanistic basis for these differences, as well as the contributions of other non-myocyte cell types and other organ systems, remains poorly understood. Comparative analyses between cardiac repair and regeneration in different animal models, and at different stages of development, are providing important insights into the evolutionary conservation of key processes driving heart regeneration. Such comparative analyses could enable the re-activation of regenerative processes in the adult human heart following myocardial infarction (MI), which is a major unmet need in the clinic. In addition, such comparative analyses are useful from an evolutionary perspective because they highlight the importance of cardiac adaptations that have occurred over hundreds of millions of years during the transition from aquatic to terrestrial life, as well as during the transition from the womb to an oxygen-rich environment at birth. Interestingly, both of these transitions are associated with a decrease in cardiac regenerative capacity and are associated with major physiological, morphological and metabolic adaptations of the cardiovascular system.^1,2^ In this review, we survey differences in cardiac regenerative capacity across the animal kingdom and at different stages of development from fish to humans. The evolution and ontogeny of cardiac morphological, physiological and metabolic adaptations will be discussed in the context of heart regeneration to provide a new perspective on the development of regenerative therapies for human heart disease.

## Overview of regeneration in the animal kingdom

Regeneration is a trait that exists within different phyla, orders and species in the animal kingdom.^3,4^ Invertebrates such as planarians, crustaceans, cnidarians, echinoderms and insects, are known to have a strong global regenerative potential. However, regenerative capacity can vary considerably even in a given order. Even among the highly regenerative planarian flatworms, which are capable of regenerating large portions of the body including the head,^5^ species with a more limited regenerative ability are found. For example, *Procotyla fluviatilis* has a restricted ability to replace missing tissues due to changes in the Wnt signalling pathway in this planarian species.^6^ Similarly, regenerative capacity is also differentially distributed in vertebrates. Newts, axolotls and zebrafish are well-known for their abilities to replace entire limbs, fins and other body parts following amputation.^7,8^ In contrast, regenerative capacity is restricted in many mammals. While regenerative capacity in non-mammalian vertebrates seems to rely on the re-activation of highly conserved developmental signalling pathways, it is currently unclear whether the lack of regenerative potential in mammals is due to an improper re-activation of these developmental pathways or whether it is due to intrinsic cellular changes that prevent the cells from responding in a regenerative manner to the developmental cues (or both). Nevertheless, the finding that evolutionarily conserved mechanisms drive regenerative processes is a strong argument to use comparative animal studies to identify pathways for organ regeneration in humans. With respect to the heart, such studies are particularly vital because MI severely impacts cardiac function and increases the likelihood of progression to heart failure, which represents a major disease burden in humans.^9^ The following section provides a comprehensive survey of non-mammalian vertebrates in which cardiac regeneration has been investigated.

## Heart regeneration in non-mammalian vertebrates

### Fishes

#### Adult zebrafish (*Danio rerio*)

**Figure 1:**
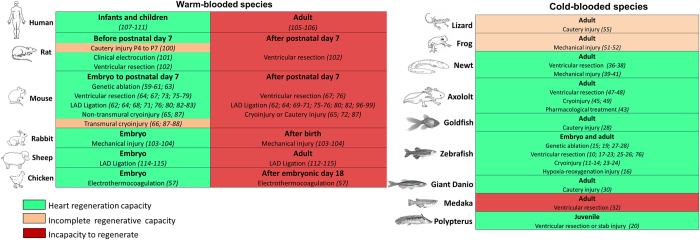
Heart regenerative capacity in warm- or cold-blooded animals. For each species, cardiac regenerative ability is indicated in green (ability to regenerate), orange (incomplete capacity to regenerate) or red (incapacity to regenerate). In each case, the approach used to induce cardiac damage and the references associated are indicated. In warm-blooded species, cardiac regeneration appears to be restricted to a defined early-developmental period during embryonic and early-neonatal life. In cold-blooded animals, six out of nine species have the ability to regenerate their heart during adult life, whereas three out of nine species show an incomplete capacity or incapacity to undergo heart regeneration.

**Figure 2:**
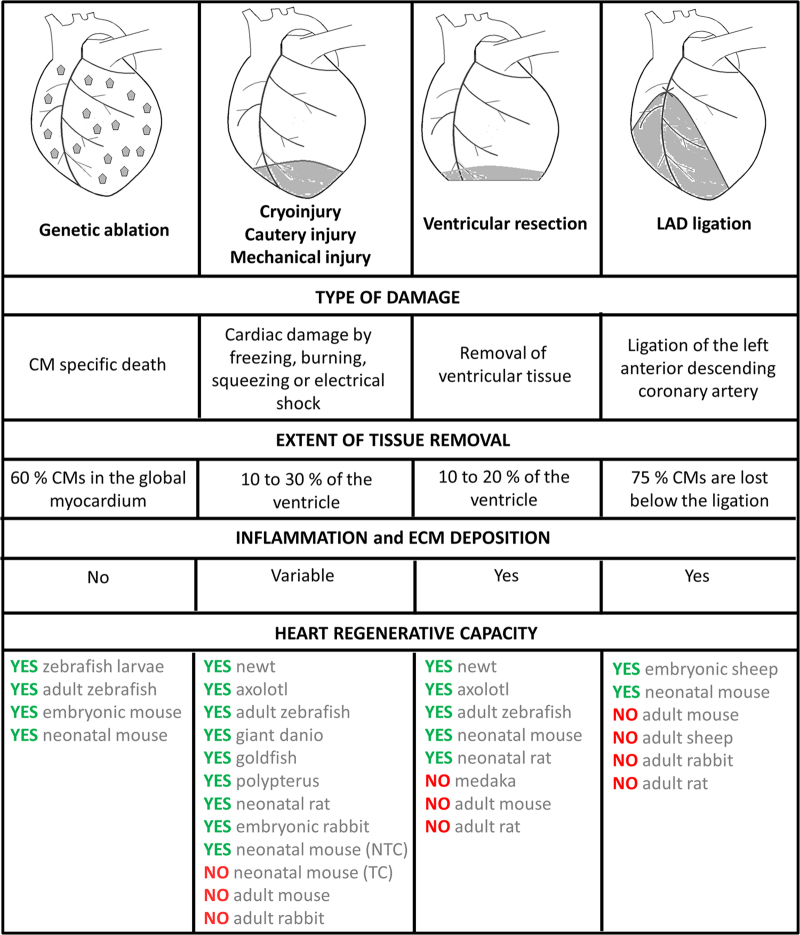
Different approaches used to study cardiac regeneration in vertebrates. The four main methodological approaches used in the literature to induce cardiac damage are indicated. For each approach, the type of injury induced, the extent of tissue removal and the extent of inflammation and ECM deposition after injury are indicated. The ability to regenerate the myocardium after the defined type of injury is indicated for the vertebrate species analysed. NTC, non-transmural cryoinjury; TC, transmural cryoinjury.

Zebrafish, a member of the Cyprinidae family of teleost fish, has the capacity to regenerate multiple tissues following injury, including the heart. There are currently four approaches used to induce myocardial damage in the adult zebrafish model. The classical and most popular model involves surgical resection of the apex of the heart (Figure 1). Using this methodology, ~20% of the cardiac ventricle is amputated and full regeneration occurs in ~60 days^10^ (Figure 2). An alternative method of cardiac injury is cryoinjury-induced myocardial ablation model. While the degree of myocardial damage and kinetics of regeneration induced by cryoinjury depend on the size of cryoprobes used and the duration of cryoinjury, this method typically results in destruction of ~15–25% of the ventricle (Figure 2). Cardiac regeneration following cryoinjury in zebrafish is much slower than apical resection with full functional recovery requiring up to 180 days.^11–14^ The third method involves the specific, non-surgical, destruction of cardiomyocytes using a genetic ablation model. This system induces the loss of up to 60% of the myocardium and zebrafish are able to mount a quick regenerative response with full recovery in 30 days^15^ (Figure 2). As opposed to surgical amputation or cryoinjury models, genetic cardiomyocyte ablation preserves endocardial and epicardial tissues and occurs in the absence of tissue destruction and clotting. The fourth method is the induction of hypoxia–reoxygenation injury in which zebrafish are subjected to transient hypoxia by reducing the oxygen saturation of water to 5% O2.^16^ After several hours of hypoxia, the authors reported evidence of cardiac oxidative stress, inflammation, cardiomyocyte apoptosis and necrosis. In the ensuing days, proliferating cardiomyocytes appear in the ventricle and a progressive restoration of cardiac structure and function is seen 30 days after the hypoxia–reoxygenation injury.^16^ While cardiac injury models in adult zebrafish differ with respect to the size of the injury, presence or absence of transient fibrotic scar, the degree of inflammation induced after damage and the timing of the regenerative response, a common thread among all of these models is the induction of a swift and robust regenerative response following cardiac injury (Figures 1 and 2).

One of the cardinal features of the cardiac regenerative response in adult zebrafish is the proliferation of pre-existing cardiomyocytes, which undergo de-differentiation, re-enter the cell cycle and migrate into the injured area following damage.^17–19^ The immediate response to cardiac injury is characterised by the secretion of developmental growth factors, which are primarily released from the epicardium, endocardium and the region adjacent to the wound border. For example, retinoic acid is released from the endocardium and epicardium following injury and acts on cardiomyocytes to promote proliferation in the sub-epicardial layers and adjacent to the wound border region.^17,20^ Similarly, bone morphogenetic protein is activated in the wound border region and stimulates cardiomyocyte proliferation following apical resection injury.^17^

Downstream of these important mitogens, several transcription factors have been identified as key mediators of zebrafish heart regeneration. A recent study has implicated NF-kB signalling in cardiac regeneration, whereby NF-kB is induced in zebrafish cardiomyocytes following cardiac injury.^21^ Inhibition of NF-kB activity impairs heart regeneration by decreasing cardiomyocyte proliferation and epicardial activation.^21^ The cardiogenic transcription factors Gata4 and Hand2 are also re-expressed in regenerating cardiomyocytes in the adult zebrafish heart, consistent with a re-activation of cardiogenic pathways during adult heart regeneration.^19,22^

A recent study assessing cardiomyocyte proliferation in the cryoinjured zebrafish heart has offered an alternative model of cardiac regeneration whereby a transient population of undifferentiated cardiomyocytes (similar to cells in the wound blastema) participate in the generation of new cardiomyocytes.^23,24^ It has been proposed that this process occurs in parallel to cardiomyocyte proliferation in this model. Furthermore, non-cardiomyocyte populations in the heart also regenerate after myocardial damage and they are required to support cardiomyocyte proliferation and remodelling.^25^ For example, the endocardium and epicardium are activated following injury and re-express developmental genes, which contribute to cardiomyocyte proliferation, angiogenesis and fibroblast differentiation.^25,26^ These studies highlight the potentially important roles of non-myocytes in the regenerative response but the precise functions of these other cell types in conferring regenerative capacity remain poorly understood.

#### Zebrafish larvae

To date, only one approach has been used to study cardiac regeneration during early-embryonic stages of zebrafish development (Figure 2). In this study, cardiomyocytes were genetically ablated in zebrafish larvae using a method that enabled cardiomyocyte-specific and temporally controlled depletion of ventricular cardiomyocytes.^27,28^ Interestingly, zebrafish embryos are able to fully regenerate the damaged myocardium in only 4 days after this type of injury but the mechanism of regeneration differs from the adult heart. The authors described a global proliferative response in the regenerating ventricle, with proliferating myocytes detected in the atria, as well as the ventricle.^27,28^ However, in addition to cardiomyocyte proliferation, genetic fate-mapping studies revealed that cardiomyocytes from the atrium were able to migrate into the damaged ventricle, proliferate and regenerate the ventricular myocardium.^28^ Cardiomyocytes from the atrium underwent an atrial-to-ventricular transdifferentiation (or reprogramming) process, which required the activation of Notch signalling in the atrial endocardium.^28^ To date, this transdifferentiation process has only been described in zebrafish larvae but this finding suggests that the same organism can employ multiple mechanisms of cardiac regeneration depending on the developmental stage.

#### Giant Danio (*Devario aequipinnatus*), Goldfish (*Carassius auratus*) and Polypterus (*Polypterus senegalus*)

The Giant Danio and Goldfish are also teleosts belonging to the Cyprinid family. To date, the regenerative response to only one type of injury, namely cauterisation using a hot probe to burn the ventricular myocardium, has been documented in these two fish models^29,30^(Figure 1). In the Giant Danio, cauterisation induces the destruction of 30% of the ventricle, whereas this mode of injury induces damage of 11% of the ventricle in Goldfish. Both of these fish mount a robust regenerative response following cardiac injury and they are able to reconstitute ~96% of the myocardium in 40–60 days. The cardiac regenerative response in the Giant Danio is similar to the regenerative response of adult zebrafish following cryoinjury and is associated with the presence of inflammatory cells, collagen deposition and the establishment of a transient fibrotic scar that is progressively replaced with new myocardial tissue.^30^ The Giant Danio is twice as large as the zebrafish and Goldfish is even bigger. As a consequence, these models may be more suitable to the development of more physiologically relevant cardiac injury models such as coronary artery ligation to induce MI, which would facilitate direct comparative studies between fishes and mammals. Thanks to their larger size, these fish models could also be useful for comparative analyses of the cardiac regenerative response to ventricular amputation or cryoinjury at different developmental stages in teleosts.

Cardiac regeneration has also been assessed in the ancient fish *P. senegalus*, which is a member of the Polypteridae family. The last common ancestor between Polypterus and teleosts lived ~400 million years ago. Ventricular resection or stab injuries were performed on the heart of juvenile Polypterus.^20^ Interestingly, 7 days after injury, cardiomyocyte proliferation was seen in the injured area.^20^ As in zebrafish, retinoic acid was released from the epicardium and endocardium after cardiac injury in Polypterus.^20^ These findings suggest that the epicardial and cardiomyocyte proliferative responses to cardiac injury are highly conserved by evolutionarily distant species capable of heart regeneration. However, it should be noted that the rates of cardiomyocyte proliferation observed in Polypterus were lower than the rates observed in zebrafish.^20^ Moreover, the true extent of cardiac regeneration in Polypterus is unclear because this study did not provide quantitative analyses of the extent of tissue removal and fibrosis after injury.

#### Medaka (*Oryzias latipes*)

Medaka is a teleost fish that is approximately the same size as zebrafish. However, medaka belongs to the Beloniformes order, which diverged from the Cypriniform order during the early Triassic around 215 million years ago.^31^ Interestingly, in contrast to zebrafish, Giant Danio and Goldfish, medaka lack cardiac regenerative ability. Mechanical resection of the apex of the heart results in removal of 20% of the medaka ventricle, which is a similar injury size to zebrafish apical resection models.^32^ After amputation, a blood clot forms at the injury site and is subsequently replaced by a fibrotic scar. The initial events following ventricular amputation in medaka are similar to the first events occurring in the zebrafish model, with the exception of a lack of epicardial activation of retinaldehyde dehydrogenase 2 (*raldh2*), which is required for synthesis of retinoic acid. Contrary to zebrafish, no difference in cardiomyocyte proliferation was seen and the collagen area expanded instead of regressing.^32^ The absence of retinoic acid signalling from the epicardium could account for the lack of regenerative capacity in medaka but further studies would be required to confirm this hypothesis.

Taken together, regeneration studies performed in teleost fish demonstrate that fish belonging to the Cyprinid family have a widely conserved capacity to regenerate the myocardium after damage whereas the medaka, an Adrianichthyidae family member, does not possess cardiac regenerative abilities (Figure 1). Therefore, it appears that cardiac regenerative capacity is not conserved across all teleost fish. More studies in others fish families are necessary to clearly understand the evolutionary basis for regenerative differences, as well as the evolutionary pressures that have driven the acquisition and maintenance of regenerative capacity in some fish and the loss of regenerative capacity in others. To this end, it would be interesting to examine the capacity for cardiac regeneration in lungfish, which represent an important evolutionary intermediate between aquatic and terrestrial vertebrates. Lungfish are known to be able to fully regenerate their fins following amputation^33^ but it is unknown whether a similar regenerative response occurs following cardiac injury.

### Amphibians

Amphibians can be separated into three orders, urodele (newt, salamander and axololt), anuran (frogs and toads) and apoda (caecilians). Urodele amphibians have a functional tail in adult life, whereas anuran amphibians lose their tail after metamorphosis and Apoda are limbless with a worm-like form. Anuran and urodele amphibians are able to regenerate several tissues and organs such as limbs, retina and nerve tissue. Moreover, they are characterised by their two different life environments because they live in water as an aquatic (e.g., axolotl) or larval (e.g., tadpole) form; or on land as a terrestrial form (e.g., frogs, salamanders and terrestrial axolotls). Neoteny, which is the maintenance of larval/aquatic characteristics throughout life, naturally occurs in urodele amphibians. So, urodele and anuran amphibians are useful models to explore the mechanisms of cardiac regeneration in aquatic and terrestrial tetrapod vertebrates.

#### Urodele amphibians

##### *Adult newts (Notophthalmus viridescens*).

The earliest studies of cardiac regeneration following resection of the adult newt heart described the presence of proliferative cardiomyocytes in the injured myocardium, but the authors concluded that newts were not able to fully regenerate because of the presence of fibrotic tissue following injury.^34,35^ However, recent studies have provided evidence that the adult newt can regenerate its heart following the removal of a lateral portion of the ventricle (5–10%) or following ventricular resection of 20% of the apex of the heart.^36–38^ Newts can also fully regenerate after mechanical injury induced by crushing the heart with repeated squeezing between forceps^39,40^ (Figures 1 and 2). The timing to complete regeneration depends on the method of injury but typically occurs within 60–90 days. Of the aforementioned injury models, mechanical crush injury is considered to be the most similar to MI because it induces the internal loss of the myocardium and does not involve removal of the endocardium.^39,40^ However, to date, more physiologically and clinically relevant models of injury such as cryoinjury or ischaemic injury have not been reported in the newt. Molecular and genetic analyses are also limited in amphibian models because of the difficulties associated with sequencing their enormous genomes, estimated to be 10^10^ bases or ~10 times the size of the human genome.^41^ However, recent transcriptional and proteomic approaches have provided some insight into the mechanisms implicated in newt cardiac regeneration.^38,41^ Interestingly, the most significant changes in terms of gene expression in the regenerating newt heart are associated with the extracellular matrix (ECM), which implicates an important role of ECM signalling in the regenerative process.^37^ Epicardial ECM deposition was reported to be associated with cardiomyocyte proliferation in this model and the authors proposed that epicardial ECM deposition guides cardiomyocyte migration to the injured area.^37^ Finally, cardiomyocyte de-differentiation and proliferation also occurs in the adult newt heart as there is a strong downregulation of sarcomeric proteins and cardiac-specific genes following injury, which are characteristic features of de-differentiated and proliferative cardiomyocytes in the injured heart.^39,42^ The upregulation of developmental transcription factors such as Hand2 and Gata4, as well as the presence of proliferating Gata4-positive cells, has also been documented in this model, thus identifying several molecular and cellular similarities to zebrafish heart regeneration.^38^

##### *Axololts (Ambystoma mexicanum* and *A. dumerilii*).

Axolotls are known to regenerate their hearts following at least three different types of injury: surgical resection of the ventricle, cryoinjury and pharmacological heart failure induced by isoproterenol administration (Figures 1 and 2). High doses of isoproterenol overstimulate the heart and induce cardiac hypertrophy, cardiomyocyte death and fibrosis.^43,44^ *A. dumerilii* is able to gradually recover cardiac function from days 5–90 after this pharmacological intervention. However, an analysis of cardiomyocyte proliferation and myocardial regeneration in this injury model has not been undertaken.^43^ In contrast, cryoinjury and resection injury induce a more well-defined structural damage to the myocardium. Following cryoinjury or ventricular resection, *A. mexicanum* has the ability to structurally and functionally regenerate within 60–90 days.^45–48^ This regenerative response is associated with the formation of a transient fibrotic scar, which is subsequently replaced by new cardiomyocytes. Epicardial cells, as well as atrial and ventricular cardiomyocytes, proliferate in response to cardiac injury in axolotl, indicating the involvement of multiple cell types in the global response to cardiac damage.^45,46,49^

#### Salamanders and post-metamorphic axolotls

To date, there are no studies analysing the capacity for heart regeneration in terrestrial urodele amphibians (i.e., salamanders and post-metamorphic axolotls). However, it is known that the efficiency of limb regeneration is low in land-phase amphibians compared with their water-phase forms. Indeed, the aquatic amphibians can fully regenerate the limb in <60 days, whereas terrestrial amphibians require 200–400 days to regenerate.^1^ It would be interesting to determine whether cardiac regenerative capacity differs in aquatic and terrestrial amphibians to understand the impact of cardiac adaptations during the transition from water to land on regenerative capacity.

#### Anuran amphibians

Unlike cardiac regeneration studies in teleosts or urodele amphibians, heart regeneration has not been rigorously examined in anuran amphibians. Although partial heart regeneration was first reported in the adult frog over 55 years ago, this conclusion was based on the presence of mitotic cardiomyocytes and appearance of poorly differentiated myofibres in histological sections, as well as restoration of rhythmic contraction.^50^ The authors subsequently concluded that incomplete regeneration occurs in the adult frog heart following mechanical injury induced by pressing or crushing the apex^50–52^(Figure 1). However, the true extent of cardiac regeneration in this model remains unclear because these earlier studies did not provide any quantitative analyses of the extent of tissue removal and regeneration or fibrosis. Interestingly, studies on limb, retina and nerve tissue regeneration in *Xenopus laevis* show that these structures can fully regenerate in tadpoles although they cannot regenerate during adult life.^53^ Thus, it will be very interesting to investigate the capacity for heart regeneration at different developmental stages in anuran amphibians.

### Diapsids (crocodiles, lizards, snakes and birds)

Lizards and snakes have a three-chambered heart, whereas crocodiles have a four-chambered heart. These models might be considered attractive models to study heart regeneration. Diapsids are able to regenerate several organs and tissues and are often considered to own an intermediate regenerative ability that is lower than fish or amphibians but higher than mammals. The ability to regenerate in diapsids differs among different species but interestingly organs containing muscle structure, such as the tails of crocodiles and lizards, are able to regenerate.^54^ By contrast, tail regeneration has never been observed in snakes.^54^ However, for unclear reasons, the analysis of heart regeneration in diapsids has been largely forgotten or ignored in the literature. Fifty years ago, a study investigated the cardiac regenerative ability of lizards after cauterisation of the ventricle. Cardiac regeneration was evaluated in histological sections and by the quantification of DNA synthesis and mitosis in cardiac nuclei within the injured area.^50,55^ Proliferative cells increased after damage but the authors concluded that regeneration was incomplete because of the presence of a mixed muscle-connective tissue scar^50^ (Figure 1). Moreover, after consumption of a large meal, the python heart increases in mass by 40% within 2–3 days. This postprandial heart growth in pythons is characterized by cardiomyocyte hypertrophy in the absence of cardiac cell proliferation, suggesting that adult snake cardiomyocytes do not proliferate in response to increased growth demands.^54,56^

Birds are warm-blooded animals and have a four-chambered heart. The cardiovascular system of birds is very efficient because it needs to meet the high metabolic demands of flight. Interestingly, analyses performed over 30 years ago in *Gallus domesticus* showed that 3- and 5-day-old chick embryos were able to mount a complete regenerative response within 7–10 days following myocardial injury induced by electrothermocoagulation^57^ (Figure 1). However, similar injuries performed in the myocardium of 18-day-old chick embryos or in newly hatched birds induced the formation of a scar in the injured area.^57^ The finding that the myocardium of 3- and 5-day-old chick embryos can regenerate is supported by the finding that sarcoma formation (i.e., tumorigenic cardiomyocyte proliferation) can be induced in the heart of 2- and 3-day-old chick embryos, whereas the hearts of 4- and 5-day-old chick embryos are more resistant to cardiac sarcomas following exposure to the same MC29 virus strain.^58^ This analysis suggests a gradual decrease in the proliferative ability of cardiac cells during chick embryonic development. Thus, as in mammals, cardiac regenerative potential in birds appears to be restricted during a brief developmental window coinciding with rapid heart growth.

## Heart regeneration and repair in mammals

Adult mammals (including humans) can regenerate damaged tissues such as skeletal muscle and large parts of the liver but they have a very limited capacity to regenerate some other organs. The heart is one of the least regenerative organs in adult mammals and there is an enormous medical need for cardiac regeneration therapies because cardiovascular disease remains the leading cause of death worldwide. Following a heart attack, myocardial ischaemia leads to a reduction in blood supply to the myocardium and induces a transient hypoxia, which causes cardiomyocyte death primarily by necrosis.^9^ An inflammatory response ensues whereby macrophages and neutrophils remove the necrotic cells and also initiate a secondary wave of cardiomyocyte death, which results in thinning of the ventricular wall. The infarcted adult heart is not able to regenerate the lost cardiomyocytes, which are replaced by a permanent fibrotic scar, which diminishes the contractile capacity of the heart. Cardiac hypertrophy and electrophysiological adaptations further contribute to the contractile demise of the heart. Once heart failure is established, the only viable treatment option is heart transplantation, which is limited by organ donor availability and complicated by the need for lifelong immunosuppression.

### Rodents and lagomorphs

#### Embryonic mice

Evidence for cardiac regeneration in embryonic mice has been inferred from genetic cardiomyocyte ablation models.^59–61^ In the study performed by Drenckhahn *et al.*,^60^ a heart-specific conditional knockout was used to inactivate a gene encoding an enzyme required for activation of mitochondrial respiration. Because this gene was located on the X chromosome, female embryonic hearts contained a mixture of 50% wild-type and 50% mutated cardiomyocytes. However, the investigators noted that the diseased tissue represented only 10% of cardiac tissue volume at birth. The authors subsequently identified a significantly reduced level of proliferation in the mutant cardiomyocytes and a significant increase in proliferation of the healthy cardiomyocytes compared with controls. Thus, they inferred that embryonic hearts might have substantial regenerative capacity due to the increased proliferative response of healthy cardiomyocytes, which can compensate for the loss of up to 50% of cardiac cells.^60^ However, this study did not directly address the cardiac regenerative response after cardiomyocyte loss. By contrast, Sturzu *et al.*^61^ recently generated chimeric embryos using a genetic cardiomyocyte ablation strategy that enabled fine-tuning of different degrees of cardiomyocyte death in the embryonic heart. After loss of 50–60% of cardiomyocytes, a significant increase in proliferation of residual cardiomyocytes was seen and these cardiomyocytes were involved in the cell replacement process. Thus, it has been shown that the embryonic myocardium is able to regenerate without evidence of cardiac hypertrophy or dysfunction.^61^ To date, the contribution of non-cardiomyocyte cells in the embryonic regeneration process has not been investigated.

#### Neonatal mice

Unlike adult mammals, several studies have reported that neonatal mammals are capable of regenerating during a brief post-natal developmental window. Cardiac regeneration has been observed in neonatal mice following resection of the ventricular apex, ligation of the left anterior descending coronary artery (LAD ligation), cryoinjury and genetic cardiomyocyte ablation^62–68^ (Figures 1 and 2). The first demonstration of cardiac regeneration in neonatal mammals involved apical resection of ~15% of the neonatal mouse ventricle.^67^ Following apical resection in 1-day-old mice, a blood clot formed at the injury site, inflammatory cells were recruited to the damaged area, a new ECM was built and cardiomyocyte proliferation became activated globally in the heart.^67^ Genetic lineage tracing studies indicated that the majority of regenerated cardiomyocytes in the neonatal mouse heart were derived from pre-existing cardiomyocytes through cell division rather than from a stem/progenitor population.^67^ C-kit+ cardiac progenitor cells have also been implicated in the generation of new cardiomyocytes following cryoinjury in the neonatal heart,^66^ although recent lineage tracing studies have cast doubt on the capacity of this cell type to differentiate into cardiomyocytes during development and following MI.^69,70^

Similarly, cardiac regeneration has also been reported following ischaemic injury induced by ligation of the LAD in neonatal mice.^62,68^ Cardiac regeneration following MI in 1-day-old mice is also associated with an early-inflammatory response, ECM deposition and robust induction of cardiomyocyte proliferation.^68^ In addition, extensive angiogenesis and revascularization are associated with cardiac regeneration in neonatal mice following MI.^68,71^ Epicardial activation was also observed in neonatal mouse hearts after cryoinjury, suggesting that the epicardial response to injury is conserved in neonatal mammals.^65^ In both the apical resection and MI models, the ability to regenerate the heart is lost by post-natal day 7,^67,68^ which coincides with the developmental period when cardiomyocytes undergo maturation and lose their ability to proliferate.^72^ However, whether maturation of other non-myocyte populations in the heart during this important developmental window also plays a role in the regulation of cardiac regenerative capacity remains poorly understood.

Although numerous studies have reproduced these findings and reinforced the robust regenerative potential of the neonatal heart following apical resection and infarction,^62,73, 74, 75, 76, 77, 78, 79, 80, 81, 82, 83^ one contradictory report warrants further discussion. In 2014, Andersen *et al.*^84^ reported that neonatal mice were incapable of regenerating following apical resection injury and the authors failed to detect any increase in proliferating cardiomyocytes following injury. Several technical issues with this study have been noted and discussed at length in the literature by others.^73,85,86^ Importantly, the study by Andersen *et al.*^84^ deviated substantially from the original methodology described by Porrello *et al.*^67^ including their method of injury, which involved mechanical clamping of the apex in addition to resection, which was subsequently shown to directly impair cardiomyocyte proliferation.^73^ Clamping of the apex also introduces variability in the resection procedure and does not allow for careful exposition of the ventricular chamber, which was used as an anatomical landmark in the original study.^65,67^ These deviations likely resulted in a much larger portion of the ventricular tissue being removed in the Andersen study. The only non-normalized values of ventricular weight presented in the Andersen paper indicated that as much as 40% of the ventricle was removed using their protocol.^84,85^ A subsequent independent follow-up study by Bryant *et al.*^73^ determined that the size of resection injury was likely a major determinant of the neonatal regenerative response following apical resection. Similarly, the size of the injury also appears to be an important determinant of the neonatal regenerative response following cryoinjury, as structural recovery of the myocardium is reported after non-transmural cryoinjury in P1 hearts,^65,87^ whereas incomplete heart regeneration occurs after transmural cryoinjury^66,87,88^ (Figure 1). Thus, there are physiological limits to the amount of damaged tissue neonatal hearts are able to regenerate. Nevertheless, these models serve as powerful tools for comparative studies of cardiac regeneration, provided that stringent methodological protocols are adhered to.

#### Adult mice

It is now accepted that adult mice undergo a low but detectable rate of cardiomyocyte renewal throughout life. Although estimates of cardiomyocyte renewal in adult mice vary, most studies indicate that the rates of cardiomyocyte turnover are very low (in the order of 1% per year).^89^ Even if this renewal enables the heart to partially replace some cardiomyocytes during normal ageing it is not sufficient to cope with the massive loss of cardiomyocytes after MI. Indeed, the rate of cardiomyocyte renewal following MI only increases to ~3% within and adjacent to the area of myocardial injury compared with sham-operated hearts.^89^ Several attempts have been made to determine the origins of this small population of cycling cardiomyocytes in the adult myocardium, which could originate from proliferation of existing cardiomyocytes or from progenitor cells, or from a combination of both.^90,91^ Genetic lineage tracing experiments have thus far provided conflicting results claiming that newly formed cardiomyocytes in the adult heart are derived from progenitor cells or cardiomyocytes depending on the methods used.^70,89,92,93^ Despite these controversies, the emerging consensus is that the rate of cardiomyocyte turnover in the adult heart is extremely low and clearly insufficient to regenerate a sufficient population of cardiomyocytes following injury. This lack of regenerative capacity of the adult heart most likely reflects the post-natal loss of cardiomyocyte proliferative capacity, which occurs during the first few weeks after birth.

Some studies have reported a capacity for adult heart regeneration in certain strains of mice, suggesting that heart regeneration might be a genetically modifiable trait in mammals. For example, the MRL mouse, which is well-known for its capacity for regeneration following ear hole puncture, was also reported to undergo cardiac regeneration after cryoinjury of the cardiac ventricle.^94,95^ However, other studies have demonstrated that both LAD ligation and cryoinjury in this model induce fibrotic scarring instead of regeneration and it has been concluded that the MRL mouse is not a suitable model for cardiac regeneration.^96–99^ Therefore, the search for genetic loci that confer enhanced cardiac regenerative potential in adult mammals continues.

#### Rats and rabbits

Cardiac regenerative capacity has also been examined in neonatal rats after cauterisation injury, electrocution and apical resection^100–102^ (Figures 1 and 2). The earliest study by Robledo^100^ was performed in P4–P7 rat hearts and used a heated wire to cauterise the neonatal heart. Robledo^100^ noted an incomplete regenerative response in 4- to 7-day-old rats and concluded that the neonatal heart was incapable of regenerating. However, Robledo^100^ likely missed the critical regenerative window (by several days) and these experiments have been revisited more recently using the apical resection procedure. After resection of 15% of the ventricle in 1-day-old rats, Zogbi *et al.*^102^ found that new cardiomyocytes were generated in the damaged area. However, collagen fibres were also present in the regenerated area, although the levels of fibrosis were much lower than when the same injury was performed on 7-day-old rats, which were characterise by larger fibrotic scars.^102^ These findings suggest that near complete regeneration occurs in the neonatal rat heart and, as in the mouse model, regenerative capacity appears to decline rapidly after birth.

Other historical studies by Soviet scientists also reported that embryonic rabbits have a transient ability to regenerate the heart.^103,104^ After mechanical trauma, foetal rabbits were able to generate new tissue similar to muscle tissue at the site of injury but they appeared to lose this regenerative ability between embryonic day 24 and birth^103,104^ (Figure 1). This regenerative phenomenon does not appear to have been revisited since these early studies in the 1970s and it is unclear whether foetal/neonatal rabbits are capable for regenerating following ischaemic injury. Therefore, rodents and lagomorphs appear to be capable of undergoing regeneration at early stages of foetal and neonatal development but this capacity declines rapidly after birth. The timing of this developmental switch also appears to be species-specific and further studies are required to more fully characterise the regenerative process in a more diverse array of species.

### Humans and other large mammals

Developmental timing is an important determinant of regenerative capacity. Therefore, species differences between rodents and large mammals with regards to the timing of cardiac maturation and cardiomyocyte cell-cycle arrest need to be carefully considered. In contrast to rodents, human cardiomyocytes begin to withdraw from the cell cycle before birth. Although one study has reported that cardiomyocyte proliferation in humans may persist and contribute to heart growth for the first 20 years of life,^105^ this finding has been recently challenged,^106^ and it appears that most human cardiomyocytes stop proliferating after the first year of life.^105,106^ Despite low levels of cardiomyocyte proliferation after birth, the human heart appears to be largely incapable of regenerating following myocardial infarction. Interestingly, in contrast to the limited regenerative capacity of the adult human heart, several case studies suggest that cardiac regeneration may occur in children and infants (Figure 1). The earliest analyses of cardiac regeneration in children were performed on post-mortem histological heart sections from children with diphtheria.^107,108^ These studies suggested that cardiac regeneration might be present during early-developmental stages in humans because of the presence of split myocardial fibres and mitotic cells observed throughout the myocardium.^107^ Moreover, there is some evidence for little or no myocardial scarring in children after corrective cardiac surgery for a rare form of congenital heart disease, anomalous left coronary artery from the pulmonary artery.^109^ A more recent study reported the case of a newborn patient with a severe myocardial infarction occurring at birth. Remarkably, the child was able to recover complete cardiac function within one and a half months.^110^ Another previous study also reported the case of a newborn with the ability to recover myocardial function 22 months after infarction.^111^ These observations suggest humans may also retain the ability to regenerate the heart during the neonatal period, as described in neonatal mice and rats. However, given the inability to perform mechanistic studies in these patients, as well as several confounding variables such as concurrent pharmacotherapy and the possibility of myocardial stunning contributing to cardiac functional recovery,^110^ further studies in humans are required to substantiate these intriguing findings.

The only other large animal model in which cardiac regenerative capacity has been assessed is the sheep, which also owns a transient capacity for heart regeneration (Figure 1). Sheep embryos between 65 and 76 days of gestation can regenerate their heart after LAD ligation but adult sheep cannot.^112–115^ The precise developmental stage when this foetal regenerative capacity is lost in sheep is unknown. In this ovine model, gene expression analysis performed after myocardial infarction shows that genes implicated in inflammation, ECM remodelling, cell death, cell cycle, cell migration and proliferation are differentially expressed between foetal and adult hearts.^115^ These findings suggest that the cardiac regenerative abilities of large mammals during early development are associated with differences in the proliferative capacity of cardiomyocytes but may also reflect differences in other cardiac cell types such as vascular cells, immune cells or fibroblasts. Moreover, differences in the timing of morphological and metabolic adaptations in small and large mammals may also contribute to the regulation of regenerative capacity.

### Marsupials and chiroptera

From an evolutionary perspective, investigation of cardiac regeneration in marsupials and chiroptera is potentially informative. While direct assessments of cardiac regeneration in these species have not yet been performed, several observations have been made with regards to the regulation of cardiomyocyte proliferation in these animals that warrant discussion. The hearts of healthy adult kangaroos are hypertrophic and have a higher aerobic capacity compared with other placental mammals,^116,117^ so understanding the timing of cardiomyocyte maturation and regeneration in kangaroos is important. Analysis of cardiomyocyte ultrastructure during development in the western grey kangaroo, *Macropus fuliginosus*, showed that maturation of the left ventricle structure occurs during neonatal growth stages in the pouch.^118^ With regards to cardiac regenerative potential, nothing has been reported in marsupial models in the literature but repair of the central nervous system and spinal cord has been described in the newborn opossum. Interestingly, this regenerative capacity diminishes by post-natal day 9 and the nervous system of older opossums cannot regenerate.^119,120^ These findings indicate that marsupials may have a higher capacity to undergo regeneration during a critical period in early post-natal development, which is similar to rodents.

With regards to chiroptera, one study has analysed DNA synthesis in cardiomyocytes, as well as cardiac metabolism, in the left ventricles of bats exposed to prolonged inversion.^121^ The gravitational stress of prolonged inversion induced sarcomere disorganisation and apoptosis during the early and mid-phases of the inversion. Interestingly, in bats exposed to prolonged inversion, cardiomyocytes were smaller and marked by the presence of dividing nuclei.^121^ Cardiac cell division and cardiomyocyte remodelling during prolonged inversion in bats suggests that the cardiac cell cycle may be under the control of mechanical loading and metabolism, which would be influenced by gravitational stress.

## Evolution and ontogeny of cardiac morphology and physiology

The evolution of a multi-chambered, high-pressure, closed circulatory system has necessitated a number of important morphological and physiological adaptations. These adaptations have occurred over millions of years of evolution during the transition from water to land, and occur over days to months during the developmental transition from foetal to post-natal life. Here, we discuss these evolutionary and developmental adaptations in the context of heart regeneration.

### Morphological adaptations

**Figure 3:**
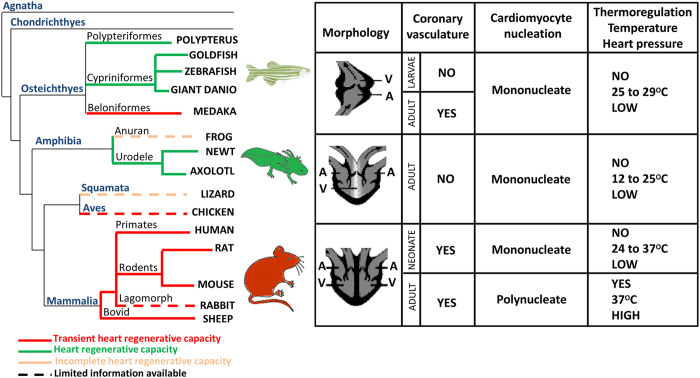
Evolution of heart regenerative capacity, heart morphology and physiology. On the evolutionary tree species in green have the capacity to regenerate the damaged myocardium, species in red have a transient regenerative ability, which is lost during adult life, and species in orange have an incomplete cardiac regenerative ability. In the table data are provided for zebrafish, axolotl or mouse models. The first column describes heart morphology, the second column indicates the presence or absence of a coronary vasculature, the third column indicates the structural maturity and nucleation status of cardiomyocytes and the fourth column indicates the capacity for thermoregulation, as well as the environmental temperature (for zebrafish and axolotl) or body temperature (for neonatal or adult mice) and heart pressure. A, atrium; V, ventricle.

Several different heart forms are found throughout the animal kingdom from the simplest heart tubes in flies to the more complex multi-chambered hearts of fish, amphibians and mammals.^122,123^ These adaptations have enabled the successful transition from water to terrestrial life. All vertebrates possess a closed and multi-chambered heart and cardiac morphogenesis progresses along a highly conserved developmental path involving formation of a linear tube, looping of the tube and finally formation of the chambers.^124^ With regards to the final morphology of the heart, fish and amphibian larvae have a two-chambered heart composed of one atrium and one ventricle. The hearts of adult amphibians are composed of three chambers, two atria and one ventricle. The adult amphibian heart is considered as an evolutionary intermediate between the two-chambered heart of fishes and the four-chambered heart of mammals, which are comprised of two atria and two ventricles.^123^ Despite differences in the final morphological appearance of the heart in adult vertebrates, the early-embryonic events that drive formation of the linear tube, looping morphogenesis and chamber formation are highly conserved and are governed by evolutionarily conserved pathways^124–129^ (Figure 3).

In addition to cardiac chamber morphology, mammals also possess a complex coronary system with arterial output and venous return. In contrast, urodele and anuran amphibians have no coronary vessels^130^ (Figure 3). The presence of coronary vessels is variable in fish. Zebrafish possess a simple coronary circulation, which develops post-hatching.^131^ The coronary arteries in the adult zebrafish heart are located in variable positions on the dorsal surface of the ventricle,^131^ which has precluded the establishment of a coronary artery ligation model to study the regenerative response to acute myocardial ischaemia. The presence of a coronary circulation may not only relate to phylogeny but might be more broadly associated with the metabolic requirements of the animal. It has been noted that the hearts of animals with low-metabolic requirements are comprised of only capillaries and veins, whereas animals with increased metabolic demands have a more complex coronary vasculature comprising arteries and veins.^132^

Given the fact that cardiac regenerative ability is observed in two-, three- and four-chambered hearts and in hearts with or without a well-developed coronary system, the formation of a multi-chambered heart does not appear to be directly correlated with the loss of cardiac regenerative capacity. Similarly, even if angiogenic responses occur after cardiac injury in zebrafish, neonatal and adult mice, these events may not be playing an essential role in determining cardiac regenerative capacity because cardiac regeneration also occurs in zebrafish larvae and amphibians, which have a very poorly developed coronary vasculature.

### Physiological adaptations

#### Pressure

The transition from aquatic to terrestrial life necessitated the development of a high-pressure closed circulatory system.^133,134^ Indeed, animals that possess regenerative potential in adult life are known to have a low-pressure circulatory system. Similarly, childbirth and childhood in mammals are associated with dramatic increases in ventricular pressure, which increases wall stress.^135^ The increased wall stress imposes a higher workload on post-natal cardiomyocytes, which have adapted by undergoing extensive structural and metabolic remodelling during early-post-natal life (see below). Although the significance of these adaptations to a high-pressure circulatory system for cardiac regeneration are still poorly understood, a recent study suggests that ventricular unloading in the adult human heart induces cardiomyocyte cell-cycle re-entry.^136^ Therefore, the evolution and development of a high-pressure circulatory system could be a key event driving ultrastructural and metabolic adaptations of the cardiomyocyte, which may have provided a more efficient pump but came at the expense of a loss of regenerative capacity. Conversely, the relief of strong gravitational demands in water may have provided a selective pressure for the evolution of regenerative mechanisms in fish.

#### Temperature

It is interesting to note that the capacity for heart regeneration in vertebrates correlates with the appearance of warm-bloodedness (Figure 1). Fishes, amphibians and neonatal rodents lack a competent thermoregulatory system. Moreover, the thermoregulatory system of newborn humans is less efficient than adults.^137^ As a consequence, their body temperature is dependent on the environmental temperature. By contrast, adult mammals and humans have developed intrinsic mechanisms to regulate their body temperature^138–140^ (Figure 3). Interestingly, analyses of limb regeneration in newts reveal a behavioural bias towards the selection of warmer environmental temperatures that facilitate regeneration. Newts exhibit certain preferences for temperatures, which can change with season or acclimation. The study by Tattersall *et al.*^141^ demonstrated that newts display a preference for warmer temperatures following amputation compared with newts prior to surgery and compared with uninjured conspecific controls. Moreover, in zebrafish, the timing to complete fin regeneration also depends on temperature.^1^ Similarly, diminishing the temperature inhibits the regeneration process in planarians.^142^ In animal models of brain injury, hypothermia seems to enhance the regeneration of brain tissue.^143^ All together these studies suggest that environmental temperature impacts regeneration but a direct analysis of the effect of temperature on cardiac regeneration has not been performed. Nevertheless, given that thermoregulating animals have a metabolic capacity that is four times more efficient than non-thermoregulating species,^2^ it is tempting to suggest that these metabolic adaptations may have also come at the expense of regeneration.

#### Hypoxia

Fish and amphibians live in an oxygen poor aquatic environment and their hearts are highly adapted to hypoxia with low-oxygen consumption rates (MO_2_=5 μmol g^−1^ h^−1^).^144^ Thus, zebrafish display a high-physiological capacity to respond to oscillations in ambient oxygen. The mammalian embryonic environment is also characterized by low-oxygen tension, which contrasts with the high-oxygen concentration that the mammalian heart is exposed to after birth. Thus, the transition from embryonic to post-natal life, as well as the transition from aquatic to terrestrial life, coincides with changes in the oxygenation of cardiomyocytes. Consequently it appears that species with cardiac regenerative capacity reside in a hypoxic environment. Indeed, hypoxia is required for induction of cardiomyocyte proliferation and heart regeneration in zebrafish.^145,146^ Recent studies in the mouse also suggest that the post-natal transition to an oxygen-rich environment may play a role in shutting down the cell cycle by activating a DNA damage checkpoint after birth.^147^ Genetic fate-mapping experiments performed in the adult mouse heart further indicate that low-metabolic activity and hypoxia are characteristics of cycling cardiomyocytes in the adult heart.^148^ Non-regenerative cardiomyocytes appear to be adapted to an oxygen-rich environment, which places different metabolic demands on the heart. As such, there appear to be important links between hypoxia, metabolism, cell-cycle arrest and regulation of regenerative capacity, which require further mechanistic interrogation.

#### Metabolism

**Figure 4:**
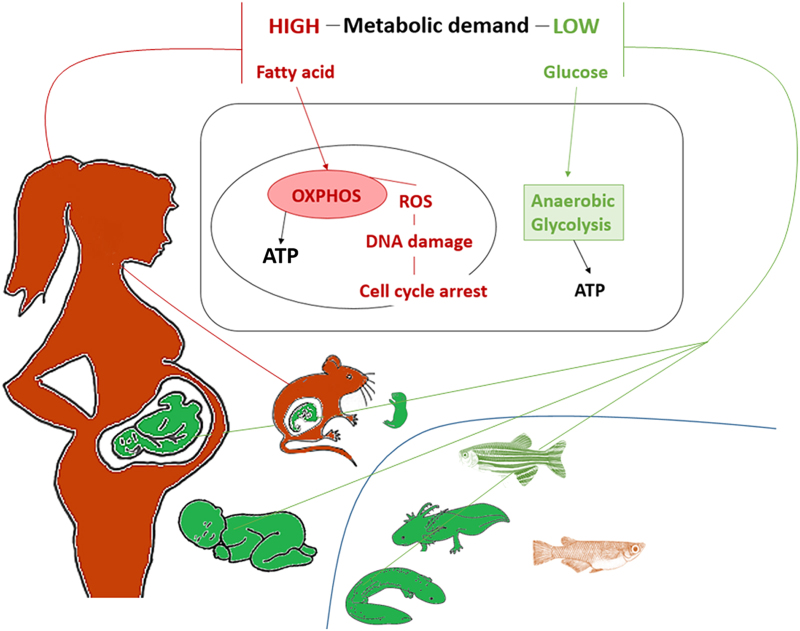
Cardiomyocyte metabolism and heart regeneration. Organisms in red are unable to regenerate the heart after cardiac damage, their energy demand is high and they mainly use oxidative phosphorylation (OXPHOS) to produce ATP. OXPHOS induces the generation of reactive oxygen species (ROS), which have been implicated in DNA damage and cell-cycle arrest. Organisms that have a low-energy demand and use anaerobic glycolysis as their main source of ATP production are shown in green.

Mammalian cardiomyocytes undergo extensive metabolic remodelling after birth to cope with the high-energy demands of post-natal life. In mice, embryonic and neonatal cardiomyocytes primarily use glycolysis as their major source of ATP^149–154^ (Figure 4). However, during the neonatal period, rodent cardiomyocytes undergo a metabolic switch and adult cardiomyocytes generate their energy via mitochondrial oxidative phosphorylation, which is more efficient than glycolysis.^151,153^ During mitochondrial oxidative phosphorylation, electron leak produces reactive oxygen species. A recent study indicates that the post-natal increase in ROS production leads to cardiomyocyte cell-cycle arrest through the activation of DNA damage response pathway^147^ (Figure 4). However, a direct causal mechanism between the metabolic switch, ROS production and cell-cycle arrest was not provided in that study and further elucidation of the mechanisms by which fatty acid oxidation contributes to post-natal cardiomyocyte maturation and/or regulation of regenerative capacity is required. Moreover, it is unclear whether cardiomyocytes from highly regenerative species such as zebrafish have a preference for glycolysis, although one study suggests there is a significantly greater capacity for oxidation of glucose than fatty acids in zebrafish.^155^ It would be interesting to determine whether high performance fish, such as tuna, which primarily utilise fatty acids as a metabolic fuel,^156^ have a similar regenerative capacity to the adult mammalian heart.

#### Structural organisation of cardiomyocytes

At the cellular level, mammalian and non-mammalian vertebrate cardiomyocytes display several differences in terms of contractile protein expression and cardiomyocyte structure. Proteomic analyses comparing zebrafish hearts with neonatal and adult mouse hearts reveal striking differences in the myofilament composition. Interestingly, zebrafish hearts are similar to neonatal mouse hearts and are characterized by an immature myofilament composition that lacks many of the structural proteins present in mature adult cardiomyocytes^157^ (Figure 3). The plasticity of zebrafish and neonatal cardiomyocytes and their capacity to dedifferentiate can be linked to their immature sarcomere composition. It is known that proliferative cardiomyocytes in the adult zebrafish and neonatal mouse heart disassemble their sarcomeres following apical resection injury.^18,22,67^ Sarcomere disassembly is an important property of proliferating cardiomyocytes.^158^ The inability of adult mammalian cardiomyocytes to proliferate could be related to their sarcomeric composition, which has likely adapted to the increased workload of terrestrial and post-natal life but could also provide a physical barrier to de-differentiation and cell-cycle re-entry. This intriguing connection between the cardiomyocyte contractile apparatus and the cell-cycle machinery remains poorly understood and it is currently unclear whether this relationship is purely associative or causal.

#### Cardiomyocyte nucleation and ploidy

Many investigators in the field have noted the association between cardiomyocyte nucleation/ploidy, proliferation and regenerative capacity. Adult zebrafish and newt cardiomyocytes, as well as embryonic mammalian cardiomyocytes are predominantly mononucleated^159^(Figure 3). In the regenerating adult newt, cardiomyocytes are mostly mononucleated in culture and the majority of these cardiomyocytes can be activated to proliferate.^42^ Interestingly, these mononucleated cardiomyocytes give rise to binucleated cardiomyocytes in their first mitosis and these new binucleated cardiomyocytes can also be activated to enter S phase in culture.^42^ Thus, the adult newt mononucleated or binucleated cardiomyocytes can proliferate *in vitro*.^42^ In mammalian models, at the end of embryogenesis and during the neonatal period, binucleation begins. In rodents, binucleation occurs during the first 2 weeks after birth^72^ and coincides with the loss of regenerative potential^68^(Figure 3). In large mammals, such as sheep and humans, binucleation occurs towards the end of gestation and prior to birth.^105,160,161^ The percentage of binucleated cardiomyocytes in the adult human heart is hotly debated and can vary from 30 to 60% of cardiomyocytes depending on the studies.^105,161^ However, one consistent finding is that human cardiomyocyte binucleation occurs primarily before birth and the rates of binucleation are lower than other species. In contrast, human cardiomyocytes undergo polyploidy after birth, which is associated with the onset of cardiomyocyte terminal differentiation.^105^ While binucleation and/or polyploidization are both associated with cardiomyocyte cell-cycle withdrawal, the physiological significance of this process is unclear. One potential explanation is that this process represents an adaptation to increase cardiomyocyte transcriptional output in response to increased energetic demands associated with the requirement for rapid growth in the post-natal period, although this theory has not been experimentally tested. This theory is, however, consistent with the rate of polyploid cardiomyocytes in birds because interspecies comparisons revealed that most cardiomyocytes in the avian myocardium are polyploid with the frequent appearance of cardiomyocytes containing three to eight nuclei.^162^ Polyploid cardiomyocytes appear soon after hatching in birds and accumulate during the first 2 weeks of post-natal development during a rapid cardiac growth phase.^162^ Interestingly, the percentage of multinucleated cells is correlated with the growth rate of the birds.^162^ Interspecies variability of cardiomyocyte ploidy levels in birds appears to be a result of changes in the cardiac functional load.^162^ However, polyploidy is also a characteristic feature of mammalian hepatocytes, which can be either mononuclear or binuclear.^163^ Even though 70% of adult hepatocytes in rodents are tetraploid, the liver still has an extraordinary capacity to regenerate from various types of injuries and hepatocytes are capable of undergoing extensive proliferation.^163,164^ Interestingly, polyploidy is dispensable for hepatic growth and regeneration in mice.^165^ Thus, binucleation and polyploidization are not required for cell-cycle withdrawal and the physiological significance of this process for regeneration still remain poorly understood. Why mammalian cardiomyocytes withdraw from the cell cycle in the perinatal period remains unclear but it is interesting to note that cardiomyopathy occurs in paediatric conditions associated with persistent markers of mitotic activity in cardiomyocytes.^166^ Interestingly, heart failure is found in affected individuals, which suggests that the restriction of cardiomyocyte proliferation is required for normal development in post-natal mammals.

#### Immune system

Several similarities between non-mammalian vertebrates and young mammals have been noted with regards to the absence of a more sophisticated immune system compared with adult mammals in terms of specificity, speed of onset and adaptive memory. For example, non-mammalian vertebrates lack specialized proteins such as immunoglobulins^167^ and the neonatal mammalian immune system has impaired pro-inflammatory functions.^168^ Urodele amphibians possess innate immunity but lack a complete adaptive immune system.^3^ Experiments performed on limb regeneration suggest that this less sophisticated adaptive immune system is involved in the regulation of regenerative ability in these species.^169^ Moreover, in anuran amphibians, tadpole tails are able to regenerate but this ability is lost during the refractory phase, which is associated with changes in the immune response to injury.^170^ Immunosuppression restored regenerative ability during the refractory period in the anuran amphibian.^170^ Interestingly, macrophages are required for appendage regeneration in zebrafish and urodele amphibians, as well as heart regeneration in neonatal mice.^63,71,169,171,172^ Macrophages are present within the infarcted area in both adult and neonatal mice in response to injury but the neonatal heart expands a population of resident cardiac macrophages, which differ from adult macrophages in terms of their origin and immunophenotype.^63,71^ Macrophages influence neovascularisation during neonatal heart regeneration but have no direct impact on cardiomyocyte proliferation in this model.^71^ Therefore, phylogenetic changes in regenerative capacity are associated with evolutionary changes in the activity of the immune system and there seems to be an inverse relationship between regenerative capacity and the development of a mature immune system. These evolutionary adaptations may have resulted from pressures that permitted the development of adaptive immune mechanisms that promoted animal survival in the face of infectious diseases but resulted in a loss of reparative potential because of excessive inflammation following tissue injury.

#### Blood clotting, inflammation and fibrosis

As previously described, depending on the animal model, heart injury can result in regeneration or permanent scar formation. Blood clotting is an early feature of the wound healing response and is associated with either regeneration or fibrotic healing depending on the animal model. In some cases, clotting factors provide signals that are required for regeneration. For example, in salamander lens regeneration and in murine liver regeneration, it appears that coagulation and other blood cell-dependant mechanisms, such as platelet activation, provide important signals for cell-cycle re-entry and regeneration.^173,174^ Interestingly, the newt lens regenerates, whereas the closely related axolotl lens cannot.^175,176^ Clotting factors, such as thrombin, have been implicated in the differential regenerative capacity of the lens in these two amphibian models.^175^ Thrombin is also known to induce cell-cycle re-entry in cultured newt myocytes.^177^ Other growth factors released from thrombocytes at the site of injury, such as platelet-derived growth factor BB isoform (Pdgf-BB), play important roles in epicardial cell function and coronary vessel formation during zebrafish heart regeneration.^178^ Therefore, blood clotting is an important early event in the cardiac regenerative response. However, blood clotting also occurs following coronary artery ligation in adult mammals, where it is associated with early-wound healing in the setting of fibrotic repair.^179^ Given that clotting is associated with both tissue repair and regeneration, it is unclear how clotting factors specifically drive regenerative processes instead of permanent scar formation.

Acute inflammation occurs after myocardial injury in regenerative and non-regenerative models. Activation of the innate immune system is one of the first events to occur after heart damage and is required for removal of dead cells and debris in the damaged tissue.^169^ Necrotic cells and debris are removed by neutrophils and macrophages at the wound site and these immune cells also release cytokines that initiate a cascade of events culminating in deposition of ECM proteins and connective scar tissue.^169^ Inflammation is considered to have a negative impact on heart regeneration by promoting fibrotic scar formation in adults.^78^ However, regenerating hearts can also react to injury by mounting an inflammatory response and depositing ECM, resulting in the formation of a transient fibrotic scar.^67,68,180^ Moreover, recent studies show a positive role for inflammation in regeneration and repair. For example, the activation of immune signals is known to facilitate skeletal muscle regeneration after injury, and, in the neonatal heart, acute cardiac inflammation is known to be important for the stimulation of angiogenesis and cardiomyocyte proliferation.^63,78,181^ The inflammatory factors that distinguish the neonatal regenerative response from the adult reparative response remain poorly defined but several recent studies point to potentially important roles for interleukin-6 and interleukin-13 signalling.^78,182^ Therefore, early-inflammatory and -fibrotic responses in the damaged myocardium *per se* do not hamper heart regeneration and further studies are required to fully elucidate how tissue inflammation can direct either permanent scar formation or heart regeneration.

## Conclusion

To date, comparative analyses devoted to understanding the regenerative potential of the heart have been performed in a dozen or so vertebrate species (Figure 3). In teleost fish, heart regeneration occurs during larval and adult life, although regenerative mechanisms differ depending on developmental stage and type of injury. For reasons that are currently unclear, some adult teleosts, such as medaka, cannot undergo cardiac regeneration. It is unclear whether cardiac regenerative ability was lost in medaka during its evolutionary divergence from zebrafish or whether zebrafish gained the genetic networks required for regeneration. In amphibians, heart regeneration occurs during aquatic life but, to date, cardiac regeneration has not been documented in terrestrial amphibians. Cardiac regeneration in mammals appears to be restricted during a defined early-developmental period during embryonic and early-neonatal life (Figure 1). The genetic and environmental causes of these evolutionary transitions over hundreds of millions of years of evolution remain poorly defined.

In most vertebrate species, cardiac regenerative potential seems to correlate with a low-metabolic state, low heart pressure, immature cardiomyocyte structure, hypoxia, an immature immune system and the inability to regulate body temperature (Figures 3 and 4). The relationship between these physiological parameters and the ability to regenerate is not well-understood. From an evolutionary perspective, it will be important to assess heart regeneration in a more diverse array of species including birds, reptiles and marsupials. Such comparative analyses would enable the identification of core evolutionarily conserved mechanisms for vertebrate heart regeneration and provide a more nuanced understanding of the role of genes and the environment in the regulation of this complex process. Taken together, cardiac regenerative ability is a highly variable trait that exists in diverse species and at different stages of development in multiple vertebrates across the animal kingdom. Understanding the evolutionary context and mechanisms that govern cardiac regenerative capacity in mammals during early stages of development would provide stronger scientific foundations for the translation of cardiac regeneration therapies into the clinic.
